# Paliação Transcateter para Tetralogia de Fallot

**DOI:** 10.36660/abc.20210735

**Published:** 2021-10-06

**Authors:** Francisco Chamié

**Affiliations:** 1 Intercat - Cardiologia Intervencionista BotafogoRJ Brasil Intercat - Cardiologia Intervencionista, Botafogo, RJ - Brasil

**Keywords:** Tetralogia de Fallot, Cianose, Cateterismo Cardíaco, Stents

A Tetralogia de Fallot (TOF) é o defeito cardíaco cianótico mais comum. A principal característica morfológica da TOF é o desalinhamento do septo infundibular. O desalinhamento do septo infundibular faz com que a aorta cavalgue o septo interventricular (dextroposição) num grande defeito do septo ventricular (DSV) e promova obstrução do trato infundibular ventricular direito. A válvula pulmonar também é estenótica, e o tronco pulmonar e as artérias são, até certo ponto, hipoplásicos. A obstrução severa ao fluxo sanguíneo pulmonar (FSP) leva a uma insaturação sistêmica e, portanto, à hipóxia prolongada.

A cirurgia cardíaca aberta é a modalidade tradicional de tratamento, aumentando o trato de saída ventricular direita (RVOT), fechando o DSV, redirecionando assim a aorta para o ventrículo esquerdo, corrigindo a anatomia cardíaca e normalizando a saturação do fluxo sistêmico.^1^

Alguns pacientes não são candidatos à cirurgia precoce devido ao peso corporal insuficiente, pequeno tamanho da artéria pulmonar (má anatomia), prematuridade, comprometimento neurológico ou defeitos associados.^2 , 3^ Nesses casos, procedimentos paliativos são necessários para aumentar a saturação sistêmica de oxigênio e o FSP, reduzindo os níveis de hipóxia. A paliação ideal seria oferecer uma fonte de fluxo sanguíneo pulmonar estável e simétrico e crescimento adequado das artérias pulmonares, sem deixar resíduos que possam prejudicar a cirurgia corretiva.

A forma mais tradicional de paliação é o Blalock-Taussig Shunt (BTS), idealizado por Alfred Blalock e Helen Taussig e realizado pela primeira vez em 1944 por Alfred Blalock.^4^ O BTS clássico consiste em anastomose da artéria subclávia direita na artéria pulmonar direita quando o arco aórtico é do lado esquerdo. Quando a aorta está do lado direito, a anastomose é realizada na artéria subclávia esquerda.

O BTS modificado usando interposição de um enxerto de tubo PTFE foi posteriormente desenvolvido nos anos 70 com o objetivo de preservar o fluxo subclávio para o braço ipsilateral.^5^ Embora eficaz, o BTS tem alguns problemas, incluindo o FSP seletivo, desenvolvimento desigual de artérias pulmonares, estenose de ramo pulmonar mediada por sutura e oclusão de shunt com hipoxemia consequente. Além disso, deve-se levar em conta que transportar um paciente gravemente doente para a sala de cirurgia é um risco em si mesmo.

Alternativas paliativas não cirúrgicas foram buscadas, e várias estratégias foram oferecidas, como valvoplastia pulmonar por balão (VPB) e implante de stent ductal ou RVOT.

O VPB é eficaz nos casos em que a principal característica obstrutiva é a válvula pulmonar, tendo eficácia reduzida quando há estenose infundibular significativa. Nesse caso, a paliação eficaz tem eficácia de curto prazo.^6^

O stent ductal é um procedimento seguro e eficaz quando realizado em centros experientes. Tem resultados comparáveis ao BTS em pacientes selecionados com FSP dependente de ductal. Glatz et al. relatam 106 pacientes tratados com stent ductal versus 251 pacientes tratados com BTS. Os desfechos compostos primários (morte ou reintervenção) foram mais comuns no grupo BTS (29,5% vs. 17%, p= 0,014) devido principalmente a reintervenções não planejadas para alívio da cianose (10,4% x 6,6%, p=0,26). Como previsto, as complicações do processo foram mais comumente encontradas no grupo BTS, embora sem significância estatística. O crescimento da artéria pulmonar no grupo de stent ductal foi maior e mais simétrico (p=0,015).^7^ As possíveis complicações do stent ductal são re-estenose intra-stent, proliferação intimal e obstrução do stent.^8^

O stent RVOT emergiu como uma técnica convincente para a paliação da TOF. O alívio da obstrução infundibular e da estenose da válvula pulmonar por implantação de stent de metal nu no RVOT pode levar a FSP estável e crescimento satisfatório das artérias pulmonares.^9 - 13^

O implante de stent RVOT melhora o fluxo pulsátil de sangue venoso sistêmico para a artéria pulmonar, melhorando a saturação de oxigênio sem declínio na pressão aórtica diastólica e perfusão coronária resultante. Uma revisão sistemática e metanálise por Ghaderian et al. mostrou alta eficácia clínica do stent RVOT na obtenção de crescimento arterial pulmonar adequado durante a paliação e obtenção de saturação adequada de oxigênio arterial. Também apresentaram baixa morbidade pós-procedimento e mortalidade após o stent RVOT e nenhuma diferença significativa nos desfechos do processo.^14^ Em pacientes pequenos e anatomias complexas, o stent RVOT permite a correção cirúrgica em um estágio posterior. A remoção do stent durante a cirurgia, embora viável, prolonga o tempo de by-pass e, na maioria dos casos, determina o uso de remendos transanulares no momento da cirurgia definitiva.^15^

Na edição atual do Arquivos Brasileiros de Cardiologia, Kupas et al.^16^ relataram seis bebês tratados pelo stent RVOT. A idade mediana no momento do implante foi de 146,5 dias e 367 dias no momento do implante e recuperação do stent, respectivamente. Quatro pacientes tiveram obstrução infundibular, e dois pacientes predominantemente tiveram uma obstrução valvar. A avaliação imediata pós-procedimento mostrou redução do gradiente sistólico de pico, aumento do tamanho das artérias pulmonares e saturação sistêmica de oxigênio. A mortalidade geral foi de 33%. Assim, embora constituindo uma série de casos muito pequenos, os autores propõem a implantação de stent em RVOT como uma opção interessante e atraente para a paliação da TOF em recém-nascidos muito doentes.^16^

A paliação transcateter pode direcionar pacientes de alto risco ao caminho para resolução completa e fisiológica do TOF. Novas técnicas no horizonte tornam o tratamento da TOF muito provável de ser realizado de forma menos invasiva, com procedimentos baseados em cateteres e/ou híbridos.^17^
