# Supplementary material for: Paliação Transcateter para Tetralogia de Fallot
Source: Arq Bras Cardiol. 2021 Oct 6;117(4):664–5. [Article in Portuguese] doi: 10.36660/abc.20210735 (PMC8528372; doi:10.36660/abc.20210735)
Supplement: Translation

# Translation: Transcatheter Palliation for Tetralogy of Fallot

**Authors:** Francisco Chamié

**Affiliations:** 1 Brazil Intercat - Cardiologia Intervencionista, Botafogo, RJ - Brazil

**Keywords:** Tetralogy of Fallot, Cyanosis, Cardiac Catheterization, Stents

The Tetralogy of Fallot (TOF) is the most common cyanotic congenital heart defect. The morphologic key feature of TOF is the malalignment of the infundibular septum. The infundibular septum malalignment makes the aorta override interventricular septum (dextroposition) over a large ventricular septal defect (VSD) and promotes obstruction to the right ventricular infundibular tract. The pulmonary valve is also stenotic, and the pulmonary trunk and arteries are, to some degree, hypoplastic. Severe obstruction to pulmonary blood flow (PBF) leads to more systemic unsaturation and, thus, prolonged hypoxia.

Open heart surgery is the traditional treatment modality, augmenting the right ventricular outflow tract (RVOT), closing the VSD, thus redirecting the aorta to the left ventricle, correcting the cardiac anatomy, and normalizing systemic flow saturation.^1^

Some patients are not candidates for early surgery due to insufficient body weight, small pulmonary artery size (bad anatomy), prematurity, neurologic impairment, or associated defects.^2 , 3^ In those cases, palliative procedures are necessary to increase PBF and systemic oxygen saturation, reducing levels of hypoxia. Ideal palliation would offer a stable and symmetric pulmonary blood flow source and adequate growth of pulmonary arteries, leaving behind no residues that can impair corrective surgery.

The most traditional form of palliation is the Blalock-Taussig Shunt (BTS), idealized by Alfred Blalock and Helen Taussig and first performed in 1944 by Alfred Blalock.^4^ Classic BTS consists of right subclavian artery anastomosis to the right pulmonary artery when the aortic arch is left-sided. When the aorta is right-sided, the anastomosis is performed in the left subclavian artery.

Modified BTS using interposition of a PTFE tube graft was later developed in the ‘70s aiming to preserve subclavian flow to the ipsilateral arm.^5^ Although effective, BTS has some issues, including selective PBF, unequal development of pulmonary arteries, suture-mediated pulmonary branch stenosis, and shunt occlusion with consequent hypoxemia. In addition, it should be considered that transporting a severely ill patient to the operating room is a risk in itself.

Non-surgical palliation alternatives were pursued, and several strategies were offered, such as pulmonary balloon valvuloplasty (PBV) and ductal or RVOT stenting.

PBV is effective in cases where the main obstructive feature is the pulmonary valve, having reduced efficacy when significant infundibular stenosis is present. In that case, effective palliation has short-term efficacy.^6^

Ductal stenting is a safe and effective procedure when performed in experienced centers. It has comparable results to BTS in selected patients with ductal-dependent PBF. Glatz et al. report on 106 patients treated with ductal stenting versus 251 patients treated with BTS. Primary composite outcomes (death or reintervention) were more common in the BTS group (29.5% vs 17%, p= 0.014) due mainly to unplanned reinterventions for cyanosis relief (10.4% x 6.6%, p=0.26). As anticipated, procedural complications were more commonly found in the BTS group, although without statistical significance. Pulmonary artery growth in the ductus stenting group was greater and more symmetric (p=0.015).^7^ Possible complications of ductal stenting are in-stent re-stenosis, intimal proliferation, and stent obstruction.^8^

RVOT stenting emerged as a compelling technique for TOF palliation. Relief of infundibular obstruction and pulmonary valve stenosis by bare-metal stent implantation in the RVOT can lead to stable PBF and satisfactory growth of pulmonary arteries.^9 - 13^

RVOT stenting improves the pulsatile flow of systemic venous blood to the pulmonary artery, improving oxygen saturation without a decline in diastolic aortic pressure and resultant coronary perfusion. A systematic review and meta-analysis by Ghaderian et al. showed high clinical efficacy of RVOT stenting in achieving adequate pulmonary arterial growth during palliation and obtaining proper arterial oxygen saturation. They also showed low post-procedure morbidity and mortality following RVOT stenting and no significant difference in procedural outcomes.^14^ In small patients and complex anatomies, RVOT stenting allows for surgical correction in a later stage. Stent removal during surgery, albeit feasible, lengthens by-pass time and, in most cases, determines the use of transannular patches at the time of definitive surgery.^15^

In the current issue of Arquivos Brasileiros de Cardiologia, Kupas et al.^16^ reported on six infants treated by RVOT stenting. The median age at the moment of the implant was 146.5 days and 367 days at the moment of stent implant and retrieval, respectively. Four patients had an infundibular obstruction, and two patients predominantly had a valvar obstruction. The immediate post-procedure evaluation showed a reduction of the peak systolic gradient, increased pulmonary arteries size and systemic oxygen saturation. Overall mortality was 33%. Thus, albeit constituting a very small case series, the authors propose stent implantation in RVOT as an interesting and appealing option for TOF palliation in very sick neonates.^16^

Transcatheter palliation may direct high-risk patients to the path for complete and physiologic resolution of TOF. New techniques on the horizon make TOF treatment very likely to be performed less invasively, with catheter-based and/or hybrid procedures.^17^
